# On Providing Multi-Level Quality of Service for Operating Rooms of the Future

**DOI:** 10.3390/s19102303

**Published:** 2019-05-18

**Authors:** Vinicius Facco Rodrigues, Rodrigo da Rosa Righi, Cristiano André da Costa, Björn Eskofier, Andreas Maier

**Affiliations:** 1 Software Innovation Lab – SOFTWARELAB, Universidade do Vale do Rio dos Sinos – Unisinos São Leopoldo 93022-718, RS, Brazil; rrrighi@unisinos.br (R.d.R.R.); cac@unisinos.br (C.A.d.C.); 2 Machine Learning and Data Analytics Lab, Friedrich-Alexander-Universität Erlangen-Nürnberg, 91054 Erlangen, Germany; bjoern.eskofier@fau.de; 3 Pattern Recognition Lab, Friedrich-Alexander-Universität Erlangen-Nürnberg, 91054 Erlangen, Germany; andreas.maier@fau.de

**Keywords:** distributed systems, health informatics, middleware, operating room, quality of service

## Abstract

The Operating Room (OR) plays an important role in delivering vital medical services to patients in hospitals. Such environments contain several medical devices, equipment, and systems producing valuable information which might be combined for biomedical and surgical workflow analysis. Considering the sensibility of data from sensors in the OR, independently of processing and network loads, the middleware that provides data from these sensors have to respect applications quality of service (QoS) demands. In an OR middleware, there are two main bottlenecks that might suffer QoS problems and, consequently, impact directly in user experience: (*i*) simultaneous user applications connecting the middleware; and (*ii*) a high number of sensors generating information from the environment. Currently, many middlewares that support QoS have been proposed by many fields; however, to the best of our knowledge, there is no research on this topic or the OR environment. OR environments are characterized by being crowded by persons and equipment, some of them of specific use in such environments, as mobile x-ray machines. Therefore, this article proposes QualiCare, an adaptable middleware model to provide multi-level QoS, improve user experience, and increase hardware utilization to middlewares in OR environments. Our main contributions are a middleware model and an orchestration engine in charge of changing the middleware behavior to guarantee performance. Results demonstrate that adapting middleware parameters on demand reduces network usage and improves resource consumption maintaining data provisioning.

## 1. Introduction

The adoption of Internet of Things (IoT) technologies in the healthcare field has increased considerably in recent years [1]. Healthcare environments constantly demand improved quality of care and smaller operational costs [2]. In particular, the Operating Room (OR) plays an important role in delivering vital medical services for patients in the hospital [3]. In an OR setting, many sensors and applications are involved in the process of generating workflow data from procedures in real time [4]. OR settings contain many sensors and applications involved in the process of monitoring procedures, which produces valuable information for data fusion and complex analysis [5,6]. However, providing data from all sensors to user applications is challenging due to the increasing variety of heterogeneous sensors which suffer from interoperability problems [1]. IoT service-based architecture is becoming trending approach which provides a layer between user applications and data from sensors to simplify data processing [1,7,8]. The service layer is able to provide such information to any application that might require it for further processing. In summary, a middleware is in charge of collecting data from sensors and delivering it to applications that previously requested it. The middleware concept defines an intermediary layer between two or more systems to mediate its integration [9,10].

**Figure 1:**
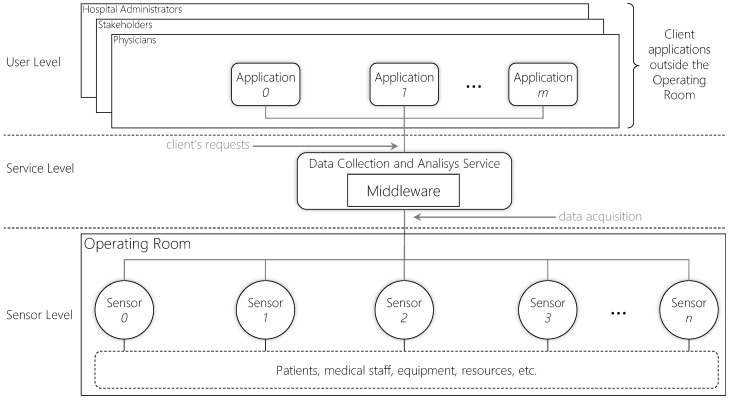
A monitored Operating Room model with a middleware acquiring and providing information in real time for user applications. The middleware is present in the service level, which can be deployed in a server or datacenter. As the number *m* user applications and *n* sensors increase, the middleware may decrease performance and consequently QoS.

Figure 1 depicts a representation of this configuration showing a scenario where multiple applications access data from several sensors in the OR. The model shows three different levels: (*i*) User, which comprises the user applications; (*ii*) Service, which encompasses the middleware responsible for integrating the other levels; and (*iii*) Sensor, which includes all sensors and devices responsible for producing data. Different kind of sensors are responsible for producing information such as medical staff positions, patients physiological parameters, equipment status, and environment conditions during procedures [11]. These sensors can be radio frequency tags and readers, camera devices, medical devices, etc. Each sensor monitors particular information, and the middleware gathers data from all available sensors providing real-time data for users. Real-time is a very important concern in such environments since saving lives is a matter of seconds in surgical procedures and, besides decreasing time response to critical situations, it is also helpful to avoid medical errors, which is a common problem in ORs [12,13,14]. It is important to point out that the middleware provides not only real-time data but also historical data, characterized by two different models: (*i*) real-time; and (*ii*) offline. The former model provides the most updated data from the OR, while the second provides data from past events. Therefore, even if there are any user applications requesting data, the middleware remains gathering data for persistence. This can be achieved, for instance, by using an external database located in the Cloud, as in [15]. Cloud platforms enable easy database scalability through the cloud elasticity feature [16].

The growing number of sensors focusing on monitoring patients, medical staff, and equipment results in a need for device data interoperability [17]. That is, the more the number of sensors the more the different information data is produced to be accessed and interpreted. Improving patients’ safety depends on the middleware capacity to acquire and provide large amounts of data in real time [17] regardless the processing and network load. This capacity depends on the middleware performance in terms of network and computing power, which must respect Quality of Service (QoS) requirements of user applications. QoS is a common concept in computing networks that consists of control mechanisms aimed to guarantee that acceptable performance levels of given service are respected. These levels correspond to measurements of several network metrics, such as packet loss, throughput, transmission delay, etc., which describe the overall performance of a service. Middlewares for OR is a novel concept with the potential to be present in the most technological ORs. Data streaming in such environments has the potential to highlight anomalies in surgery workflows and support medical decisions. If the middleware fails to deliver data at some moment violating QoS requirements, these anomalies might go unnoticed. In this context, to maintain a desirable QoS levels for real-time applications that consume data from the surgical workflow is challenging since the number of sensors and user applications might dynamically change. For instance, in a surgical procedures there are many people are involved in the process which might go in or out the OR at any time. In these situations, wearable motion and indoor location sensors, such as radio-frequency tags, accelerometers, gyroscopes, and magnetometers, are detected only when the subject is within the room. The growth of the number of sensors and devices in the OR increases the complexity of real-time information monitoring since the more the number of sensors the higher the amount of information that the OR middleware must handle simultaneously. Besides handling network connections to sensors at the sensor level, the middleware also must manage user applications requests in the User level, which demand real-time data. Therefore, it is required that an OR middleware is capable of identifying and handling such situations to keep a certain level of QoS, not impacting in the user experience.

Currently, most studies aiming at providing medical systems for ORs focus on describing architectures for data monitoring [18,19,20,21,22,23,24,25,26,27,28]. However, they do not address QoS mechanisms in their solutions, which, to the best of our knowledge, indicates a lack of studies focusing on this significant topic for patient safety. Therefore, to the best of our knowledge, there is no current research that explores QoS for OR middlewares. The current strategies focus mainly on developing monitoring systems for OR not addressing QoS. This highlights a knowledge gap that is a significant topic for patient safety. In this context, this article presents the QualiCare model, which aims to provide multi-level QoS for OR middlewares in both User and Sensor levels. The scientific contributions of this research are twofold:

- a model of an adaptable QoS-enabled middleware for OR;
- an orchestration engine to provide different services for user applications and sensors.

The model multi-level approach provides services to tackle QoS violations for both user applications at the User level and hardware devices in the Sensor level. QualiCare is an adaptable middleware model to provide multi-level QoS, improve user experience, and increase hardware utilization to middlewares in OR environments. The main focus is to guarantee that the middleware respects QoS levels by improving network and computing performance at both Sensor and User levels. The model proposes the combination of different methods to offer such features. It provides a manager module which controls QoS levels by monitoring different metrics from the sensors and middleware modules. The manager allows the parametrization of thresholds for different metrics as input, and it automatically performs configuration adjustments in all middleware modules through a reactive rule-based strategy in a periodic monitoring fashion.

The remainder of the article is structured as follows. Section 2 introduces important concepts related to this study and Section 3 describes the literature review. Section 4 and Section 5 present the core ideas of this document. The former introduces the design decisions and the architecture of the model The later presents the QoS model and strategies that QualiCare proposes. Then, Section 6 presents the preliminary results of the first experiments. Finally, Section 7 describes some limitations of the study and Section 8 presents the final remarks.

## 2. Background

Distributed system environments are characterized by resource sharing among many users and applications [29,30]. These systems rely on networked connections for communication, which may suffer instabilities and consequently impact on performance. QoS specifications define a set of parameters for different components in a distributed system [31,32]. QoS strategies are employed to guarantee that distributed systems services maintain a certain level of quality avoiding problems [29,30]. According to Wang [33], QoS is “the capability to provide resource assurance and service differentiation in a network”. In the Internet, for instance, there are many QoS requirements, including for real-time applications [34,35]. Their requirements define acceptable levels for metrics related to network, such as latency, jitter, and transfer rate. The transfer rate metric measures the amount of information that is possible to transmit in a given time interval. In general, to this time interval is considered the amount of one second and the information grain varies. Latency, in turn, is the time interval between the cause and effect of an action. More specifically, in computer networks the latency is the time interval between a sender dispatching a packet and the receiver receiving it. High latencies cause high response time of network systems and can deteriorate the quality of an online service, such as video streaming in real time.

In computer networks, jitter measures the time delay between the arrival of two consecutive packets. As in distributed networks, routers might transmit different packets by different routes, and the receiver may face variations in the packet inter-arrival time due to the disparities of the paths which can vary in number of hops and network congestion load. Such a phenomenon causes variations in the data flow arrival according to the network load and, depending on the application, strategies have to be implemented to mitigate the problem. For instance, applications that transmit and process video and voice data require the packets to arrive in a regular pace. In this scenario, packets which do not arrive in an expected time have to be discarded or the receiver must use a jitter buffer to store temporally incoming packets to smooth the arrival times.

According to Shin and Ramanathan [36], real-time systems are characterized by three main components: (*i*) time; (*ii*) reliability; and (*iii*) environment. The ”time“ is the most important factor real-time systems must control. Cooperating tasks are required to be completed within a given deadline, otherwise the computation may be compromised. In addition, reliability is required since system failures might cause catastrophes and even loss of lives. Finally, the environment under which the system operates is also important since physical events trigger the system to perform tasks and process these events. Real-time systems are classified depending on the consequences of meeting or not a deadline: hard; firm; and soft. Not meeting a hard deadline leads to catastrophic situations, for instance, in a crash of a flight due to delays in the aircraft system to computes readings of flight sensors. In the case of firm deadlines, the produced results are useless after the deadline but do not cause any harmful situation. In turn, not respecting soft deadlines produces useful results that decrease in quality as the time goes by. However, like firm deadlines, it does not cause catastrophic situations. In particular, the real-time term we are using in OR environments refers to soft real-time definition since occasional delays in the data delivery do not trigger catastrophic situations.

## 3. Related Work

The literature review in this study adopts the principles of systematic reviews [37] to achieve reproducibility and high-quality results. Its goal is to provide an overview of OR middleware strategies. The *scope* of the literature search encompasses the selection of literature databases. It is narrowed to sources that: (*i*) index articles from relevant conferences and journals from Computer Science and Medicine; and (*ii*) include a broad selection of venues to maximize the number of returned articles. Based on these criteria, the following five databases are queried: *IEEE Xplore* (https://ieeexplore.ieee.org/); *Google Scholar* (https://scholar.google.com.br/); *PubMed* (https://www.ncbi.nlm.nih.gov/pubmed/); *ScienceDirect* (https://www.sciencedirect.com/); *Springer Link* (https://link.springer.com/). Real-time, safety, and cost are highly related to the OR, therefore, our focus is to evaluate the state-of-the-art that targets ORs. Thus, to limit the scope of the article search strategy, the search string is defined as follows considering the OR term: “operating room” AND “middleware”. The combination of these strings as a search string to be used in the target databases represent the inclusion criteria. The main goal is to hit all kind of studies that in some way approach middleware in OR environments. The raw literature corpus from the inclusion criteria contains a set of 1005 articles. The final set of studies is selected through exclusion criteria applied to the raw corpus. These criteria are formed by the following removal filters:

- Removal filter I - Duplicate removal: The remaining studies from individual databases were grouped and duplicates were eliminated;
- Removal filter II - Title and abstract review: The title and abstract from each study are reviewed and those that do not address sensing technologies applied to healthcare monitoring are removed;
- Removal filter III - Year analysis: We are considering only publications within the last 10 years.

The literature corpus after application of all filters includes 31 articles. The contents of these studies are further analyzed to identify the main focus of the studies, which are:

1. Patient monitoring: [38,39,40,41,42];
2. Device integration and data interoperability: [43,44,45,46,47,48,49];
3. Smart hospitals: [50,51,52];
4. Operating Room monitoring (Intelligent OR): [18,19,20,21,22,23,24,25,26,27,28];
5. QoS-Aware Middlewares: [53,54,55,56,57].

Table 1 presents specific details from each one of them. In particular, the last two sections group the studies high related to the focus of the current research. The “Operating Room monitoring (Intelligent OR)” group presents studies that demonstrate attempts on employing tracking technologies in surgeries to improve procedures. Basically, they focus mainly on two different goals: (*i*) activity recognition [18,19,20]; and (*ii*) personnel and equipment tracking [20,21,22,23,24,25,26,27,28]. The former group is composed of articles focusing on identifying the actions, which medical staff perform during surgeries. In turn, the latter group is composed of studies employing Real-Time Location Systems (RTLS) to track the location of people and equipment present in surgeries. Both sets of strategies have the workflow monitoring as a primary goal, employing different procedures and technologies.

Analyzing the technologies employed by the studies in the group 4,RFID (Radio Frequency Identification) emerges as a common strategy present in more than half of the articles. Active RFID tags are most used since they produce accurate readings. These strategies employ tags for identification purposes and to track the location of people and equipment to improve efficiency and avoid medical errors. Besides RFID, Computer Vision techniques are present in 18% of the studies. In particular, Vaccarella et al. [21] focus on robotic systems for neurosurgery in which they integrate RTLS systems and Computer Vision techniques in real time. Regardless the technology, the majority of studies present concerns about real-time issues.

On the other hand, the “QoS-Aware Middlewares” group presents studies that focus on QoS strategies fo IoT middlewares. Rausch et al. [53] propose a solution to improve QoS in MQTT middlewares. Their strategy consists of migrating clients connections of Edge Computing applications to closer brokers. In the studies [55] and [54], the authors propose the addition of QoS management modules. The strategy adds a communication layer on top of protocols such asHTTP (Hypertext Transfer Protocol), COaP (Constrained Application Protocol) and MQTT (Message Queuing Telemetry Transport). Shi et al. [56] propose a Software-Defined Networking (SDN) controlling method to deliver different QoS levels for different clients. The solution is composed by a controller nodes that monitor and manage OpenFlow switches to configure its queue priority. Finally, Ghanbari et al. [57] present a systematic literature review focusing resource allocation in IoT. The authors demonstrate the different resource allocation strategies in the IoT field showing they belief that in the future self-adaption might become a trend for IoT systems.

By analyzing the aforementioned studies, it is possible to point out two open issues: (*i*) they do not propose specific middlewares for OR with multi-sensors; and (*ii*) lack of QoS strategies on middlewares for ORs. First, although a total of 11 articles focus the OR, the authors do not propose specific middlewares for OR. Instead, they present efforts on employing some different kind of technologies to monitor surgeries in the OR. Second, five recent studies focus on strategies for QoS middlewares or resource allocation in the IoT field. These studies present strategies focused mainly on a specific layer of the middleware, and are highly concerned in network management to decrease latency. This landscape depicts a lack of studies focusing specifically on providing QoS at both user and sensor levels of time critical middlewares. Therefore, the current document focuses on this gap by proposing its main contribution which is an adaptable middleware model to provide multi-level QoS on OR. The proposed model seeks to define the architecture and strategies necessary to guarantee QoS for user applications, and for hardware sensors. The major challenge relies on which strategies to apply and how to do it taking into account a large number of user application connections.

## 4. QualiCare Model

This article focuses on the gap presented in the previous section by proposing as main contribution an adaptable middleware model to provide multi-level QoS on OR. The proposed model seeks to define the architecture and strategies necessary to guarantee QoS for user applications, and for hardware sensors. The major challenge relies on employing different strategies considering a large number of sensors and applications. Our main focus is on QoS for hybrid OR, which are composed by a multi-disciplinary team, however our middleware can be seen as generic since it works with requirements related to CPU and I/O [58]. Thus, the system is used on demands that require QoS for these resources, requiring very low latency rates, cadence, and jitter. Therefore, the Sensor level can be seen in a generic form with data flow incoming from physical sensors, which can be a database, file stream, etc. The User level, in turn, presents applications that either request data defining their QoS requirements or, knowing the application protocol, the middleware already has standardized QoS for them. In other words, there are two types of QoS characterization: (*i*) pre-defined by the application; (*ii*) on-the-fly defined by the middleware. For instance, a video processing application may requests video frames from the middleware defining its required frames per second (FPS). On more example is an application that only needs data for real-time feedback in a dashboard interface.

Qualicare acts collecting data from sensors, storing it, and delivering it to user applications meeting QoS requirements. These requirements consists of a set of thresholds for specific metrics, called QoS metrics, that measures from them must be respected. Its main characteristic is its ability to provide QoS for both user applications and sensors regardless the system load. QualiCare provides strategies to meet soft real-time requirements, including a Manager module, which is in charge of monitoring and adaptation tasks to ensure QoS levels. Real-time is important since the capabilities of medical systems to produce data in real time enables the detection of critical situations [59]. The faster these situations are detected the higher the chances to avoid them.

### 4.1. Design Decisions

The model has two main actors involved in the production and consumption of data, respectively: (*i*) sensors; and (*ii*) user applications. QualiCare provides services to meet QoS requirements by monitoring different metrics in both sensors and user applications levels. In the user level, applications that consume data from the middleware define QoS requirements that the middleware must respect. If the user does not provide its requirements, QualiCare sets default requirements depending on the data the application requested. On the other hand, at the sensors level, the middleware defines requirements to guarantee data acquisition even if there are no user applications.

**Figure 2:**
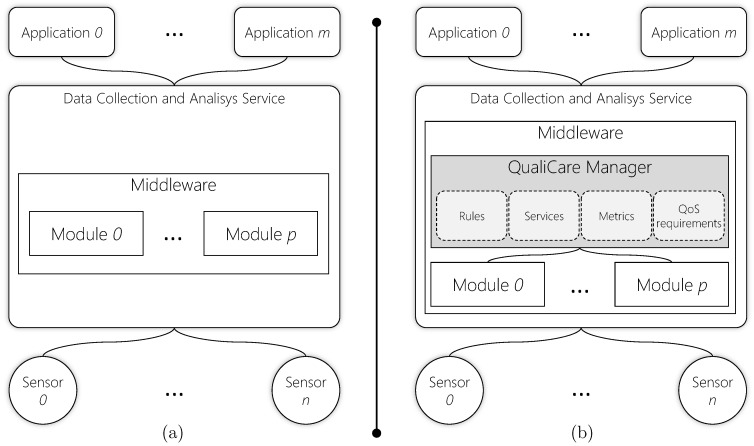
OR middleware general ideas: (**a**) default approaches; and (**b**) QualiCare main idea.

Figure 2 illustrates the QualiCare idea in comparison to a default middleware without QoS support. In each level, QualiCare monitors specifics metrics related to response time, sampling rate, and latency. Based on the results of such monitoring, QualiCare adds or removes services individually to user applications or sensors to meet the QoS requirements. This process consists of a set of threshold-based rules strategy. QualiCare Manager compares measures from metrics to lower and upper thresholds, which indicate metrics that are violating specific parameters. According to them, the decision process takes actions to adapt the middleware.

### 4.2. Architecture

**Figure 3:**
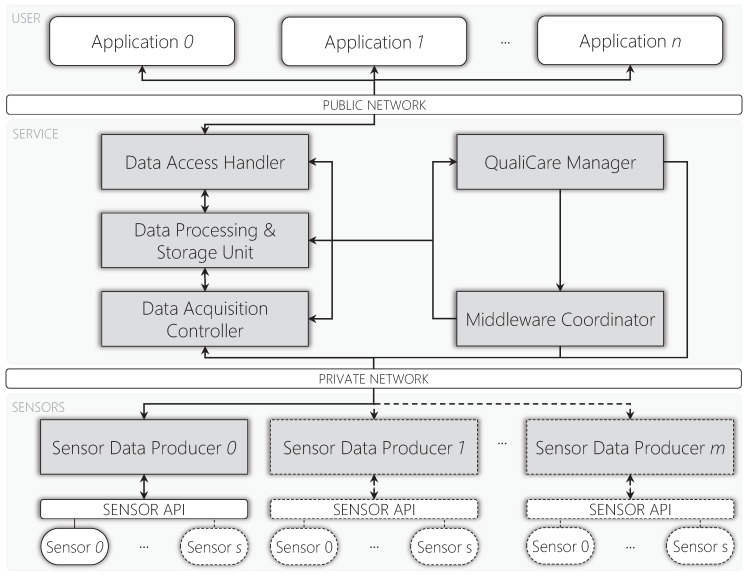
QualiCare architecture presenting the middleware in the Service level and additional modules in the Sensor level inside the OR. Arrows represent the communication direction in the following form: (*i*) application to middleware; (*ii*) middleware to applications; (*iii*) middleware to sensors; (*iv*) sensors to middleware; and (*v*) middleware components to middleware components. Items *i* to *iv* regard the sensor data flow, while item *v* regards configuration and metrics transmission.

Figure 3 depicts QualiCare architecture highlighting three different layers: (*i*) User; (*ii*) Service; and (*iii*) Sensor. User applications which consume data from the middleware compose the User layer. The components that extract and manage data compose the Sensor and Service layers, respectively. Lines connecting components represent reliable channels for data exchange or control messages. Additionally, arrows represent the sensor data flow direction. Gray boxes represent components the model provides, and the remaining white forms represent physical sensors, APIs (Application Programming Interface), and user applications. Additionally, communication between Service and Sensor layers must be in a private wired network for two main reasons: (*i*) to improve performance; and (*ii*) due to security issues since OR data is sensitive.

**Figure 4:**
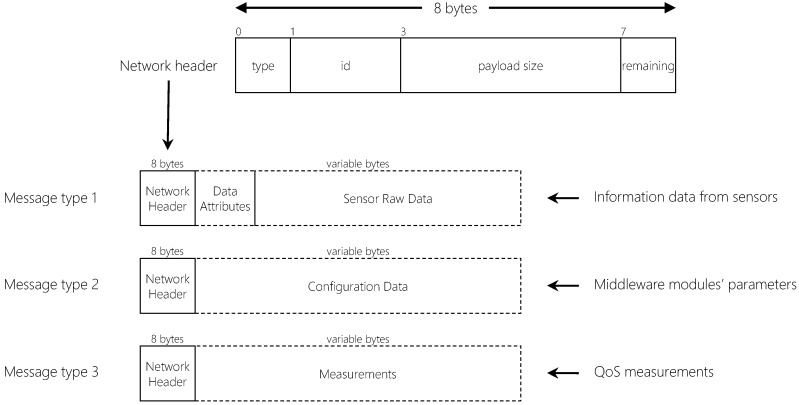
QualiCare message types and its contents. All messages use the same network header, which identifies the packets.

There are three different messages QualiCare modules might transmit between them: (*i*) configuration data; (*ii*) metrics measurements; and (*iii*) sensor data. The transmission occurs through TCP/IP messages including an 8-byte network header which identifies the messages (details in Table 2), and a variable payload that contains one of the three data. Figure 4 depicts the fields of the network header and the composition of each type of message. While the header is fixed for all messages, the payload of each one is different depending on the message type. Metric measurements messages (type 3) contain readings of metrics from modules. The QualiCare Manager consumes this type of message for QoS monitoring purposes. These values are used to evaluate the status of the middleware and monitor QoS violations. Based on that, adaptations are performed by QualiCare to guarantee QoS. In turn, configuration data messages (type 2) contain module configurations and might be used for two reasons. First, to check the current configuration of a specific module. Second, to change the parameters of the modules, which the Middleware Coordinator is the only module allowed to do. Finally, sensor data messages (type 1) represent the central information that QualiCare modules transmit, which contains the data extracted from physical sensors. This information varies depending on the sensor, which can produce different types of data depending on the sensor. For instance, a temperature sensor produce a float value corresponding the room temperature while a RTLS produces two or three integer values corresponding the 2D or 3D position of a given tag in the environment.

QualiCare architecture is composed by six different components which will be discussed in the next sections in details: (*i*) Sensor Data Producer; (*ii*) Data Acquisition Controller; (*iii*) Data Processing & Storage Unit; (*iv*) Data Access Handler; (*v*) Middleware Coordinator; and (*vi*) QualiCare Manager.

#### 4.2.1. Sensor Data Producer

In particular, the Sensor Data Producer component might have from 1 to *m* instances running in the architecture. This component extracts information directly from 1 to *s* sensors using their APIs, respecting a specific sampling rate. When running the first time, the process detects the sensors by testing the implemented APIs, and tries to reach the physical sensors using a pre-defined configuration. It includes the reachable sensors in its monitoring list, and the unreachable ones it keeps trying to reach them in periodic observations. To produce data from sensors, it extracts raw data respecting a sampling rate and builds a package with several pieces of information that characterize a sensor data. The module generates a single sensor data package for each sensor returned from the API. For instance, several RTLS tags might be acquired through the RTLS middleware API at once. The API always returns the available tags in the environment when requested.

By transforming different types of information provided by different physical sensors, this module transforms all data to a common type of information that can be interpreted by all modules without needing to implement their APIs. It allows data interoperability between the modules and between the middleware and user applications that request these data. This process consists of gathering information from physical hardware sensors through either their API or a provided service by the vendor, and transforming it into a byte array that can be transmitted over network or stored in the disc or database. The module packs the byte array in a data structure, called sensor data package, which contains six specific attributes: (*i*) sensor ID; (*ii*) device ID; (*iii*) data producer ID; (*iv*) sample counter; (*v*) timestamp; and (*vi*) type. Table 3 organizes the details of each one of them briefly. The Sensor ID, Device ID, and Data Producer ID fields identify the source of the sensor data. Sensor Data Producer instances might extract data from different physical sensors. Therefore, this set of IDs identify the sources individually. The Sample Counter defines the sample sequence of the sensor data, and the Timestamp is the instant of time that the data was extracted from the physical sensor. Finally, the Type defines the kind of data, which can be, for instance, a sample of the room temperature or the heart rate of a patient. Jointly to these fields, the raw sensor information data (byte array) is attached, composing a sensor data package.

#### 4.2.2. Data Acquisition Controller

The Data Acquisition Controller component receives sensor data packages from all Sensor Data Producers respecting specific parameters for each Sensor Data Producer, which are defined in a JSON-like configuration file. This file contains a list of tuples defining the network address and connection port for each Sensor Data Producer, the sampling rate per second, and the type of data to be acquired. It dispatches *m* threads, one for each Sensor Data Producer, which establish a TCP/IP (Transmission Control Protocol/Internet Protocol) connection using sockets. Once the connection is established, the thread starts a new thread to send sample data requests of given type mask at each time interval. The time interval is defined by dividing 1000 ms (respective to 1 s) by the sampling rate parameter, and using the result as sleep time between intervals. The data type mask is an enumeration value for different data types which can be extended for new types of data according to the available type of sensors. This parameter is important since a given Sensor Data Producer instance might extract information from more than one types of sensors. Therefore, the data type mask defines from which sensor(s) the request aims to acquire data.

Sensor data replies received by each thread are stored in a shared buffer to be accessed by the Data Processing and Storage Unit component. The thread responsible for establishing connection with the Sensor Data Producer, after dispatching the data request thread, starts to read replies in the socket. When a data replies arrives, it removes the data from the socket, populates the buffer, and triggers a signal to the Data Processing and Storage Unit informing that there is new sensor data in the buffer. Each thread has its own buffer so that write operations are performed only by one process.

#### 4.2.3. Data Processing & Storage Unit

This module is responsible for storing each new sensor data package in a database and to make it available to the Data Access Handler. The sensor data is converted to a JSON (JavaScript Object Notation) string so that it is possible to use the full string for NoSQL-like (Non Structured Query Language) databases. We opted by NoSQL systems due to its prevailing adoption in Big Data environments [60]. NoSQL distributed systems present advantages to IoT systems due to its capacity of scalability and storing multiple data types that can change over time [61]. Besides storing data in the database, the component provides sensor data in two models: (*i*) real-time; and (*ii*) offline. For the first case, each new available reading of a sensor data provided by the Data Acquisition Controller is made available to the Data Access Handler so that it can be send to user applications as soon as possible. For the second, the module retrieves data from a database according to request parameters provided by the Data Access Handler. The module employs data filtering and aggregation strategies to improve the middleware performance. It accepts all fields presented in Table 3, and uses it as filters in the database to reply the request. Besides, repeated sensor data information, in which only the Timestamp changes, are transformed to only one sensor data information to avoid transmitting duplicated data.

#### 4.2.4. Data Access Handler

The Data Access Handler component manages user application connections and provides sensor data to them. It provides a web service interface which implements two different HTTP methods that the applications can use to access data: (*i*) REST (Representational State Transfer) API for retrieving offline data; and (*ii*) MQTT API for real-time data. Applications have to supply their requests with filtering parameters and QoS requirements in terms of accepted delay to reply the request and accepted latency from the time the data is extracted to the time the request is replied. Through the REST API, the module provides a set of HTTP methods the application might call to access the data. The methods receive, through the calls, the filtering parameters, which are sensor data fields, and replies a JSON-like string to the application. The MQTT API, in turn, provides topics for each physical sensor so that applications can subscribe to receive data. At each new sensor data information available, the Data Access Handler module publishes it in the respective topic.

#### 4.2.5. Middleware Coordinator

The Middleware Coordinator has administrative assignments mainly concerned to components parametrization. This component has access to change configurations of all components in the architecture, except the QualiCare Manager. For instance, it is possible to change the sampling rate per second a specific Sensor Data Producer extracts from a physical sensor. Another example regards network and compression configurations, such as TCP port to listen for connections and the activation or not of compressing algorithms in the data. The Coordinator does not request or transmit any sensor data. Instead, it acquires components configurations being able to modify them. Therefore, this is the only module capable of changing configurations of the middleware. These operations are performed through messages type 2 (see Figure 4). The Coordinator has its own configuration file that defines all available modules and their network connection information. In addition, the Coordinator configuration file also has the configurations for each module so that, when the Coordinator starts, it updates the configurations of all modules sending a message type 2. After this process the Coordinator starts listening network connections. The module is able to receive message type 2 from the QualiCare Manager and it interprets the messages in two different ways: (*i*) if the message payload is the size of 0 bytes, then the module replies the message with the current configuration off all modules; (*ii*) if the message has a payload > 0, then the payload must be a configuration file in a JSON string of a given module that must be updated.

#### 4.2.6. QualiCare Manager

Finally, the QualiCare Manager is the main component being responsible for managing QoS by providing modifications in the Service and Sensor levels. The Manager acquires measures from defined QoS metrics from all components and applies algorithms to these data to verify if modifications are needed. The module collects the measures all metrics from each module through messages type 3 (see Figure 4). To request measures, the module sends a message with payload size of 0 bytes. All modules reply messages type 3 with the measures of all metrics it generates as a JSON string in the payload field. Therefore, the Manager receives the measures to apply its algorithms in the monitoring process. In addition, the module is able to change parameters from all modules through messages type 2 which it sends to the Middleware Coordinator. Section 5.4 describes each metric and introduces the QualiCare Manager in more detail.

## 5. Quality of Service Model

QualiCare is designed as a closed feedback-loop architecture [62], involving two main components: the QualiCare Manager and the OR middleware. Control theory is an engineering and mathematics branch focused on dynamical systems behavior, and how they are affected by feedback [63]. Therefore, service provisioning decision should be made based on the system performance according to applications requirements. QualiCare Manager presents three main functions which characterize control systems: a sensor to acquire monitoring data, a controller to evaluate measurements, and an actuator to provide services.

**Figure 5:**
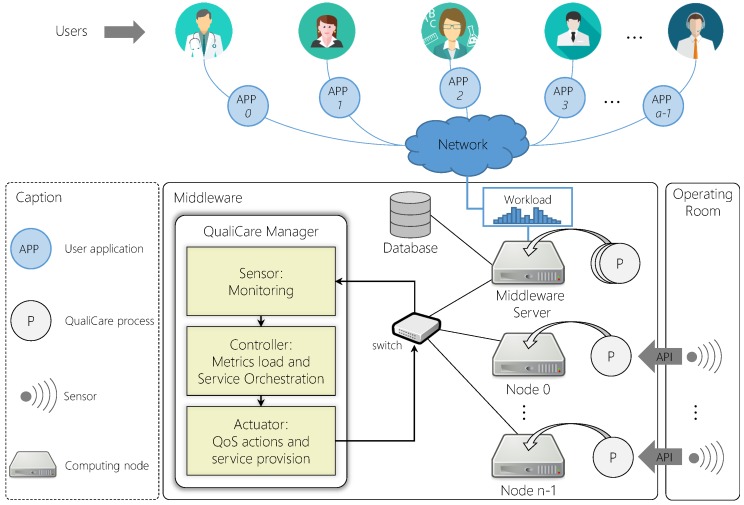
QualiCare closed-loop model with a Manager in charge of monitoring and adapting the middleware according to the workload. At the user perspective, *a* denotes the number of user applications. At the middleware perspective, *n* denotes the number of nodes running a QualiCare process that acquires data from sensors in the OR.

Figure 5 illustrates the architecture components and their organization showing the main control tasks of the QualiCare Manager. Each middleware component is represented as a Qualicare process, which can be distributed among computing nodes in a cluster within the hospital facilities or in a single server. It must be running only one instance of the Manger process to avoid concurrence of operations performed in the middleware resources. Otherwise, the same operation may be required to the Coordinator more than once. Furthermore, opposite operations might be required to the Coordinator in a short period of time if more than one Manager is running with different configurations. The Manager has access to each Qualicare process regardless of their locations, either among servers or clusters. The architecture is composed by a server and *n* nodes in which the QualiCare processes run performing the roles depicted in the previous Figure 3. The service provisioning is obtained by an orchestration model (detailed in Section 5.5), which evaluates a series of QoS metrics and defines the set of services for each component of the middleware.

### 5.1. QoS Taxonomy

In the OR scope, there are two different levels to which QualiCare taxonomy specifies QoS parameters. User parameters influence the final user perception of the performance of the middleware. These parameters are specifically related to the real-time data consumption flow by user applications. On the other hand, Sensor parameters impact the middleware data acquisition for both persistence operations and user requests. Data consumption is a continuous task, which the middleware performs for data persistence. Considering that user requests may consume data from past events (historical data), it is necessary to ensure that the middleware is able to acquire sufficient data from all sources for persistence. Lacking data in some periods of time might impact critically in the workflow analysis, making it impossible in those periods. Therefore, Sensor parameters are critical to guarantee that the middleware is able to provide both real-time and no real-time data.

**Figure 6:**
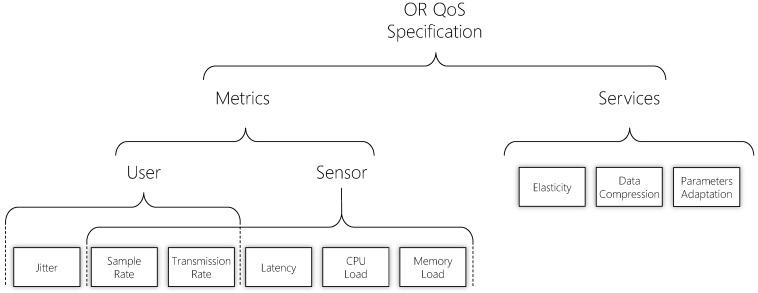
QualiCare QoS taxonomy presenting its the metrics and services.

Figure 6 depicts QualiCare QoS taxonomy presenting the metrics and services that QualiCare provides. Metrics are observed values that support the decision-making process. Services refer to methods available to tackle QoS situations based on the monitored metrics. These services impact directly in the metrics’ measures, that is, providing services changes the results of the metrics. Therefore, QualiCare Manager is in charge of monitoring such metrics and applying a suitable service to address problems. The Manager can combine a metric with one or more services or vice versa. A combination of metrics and services forms the QoS parameters.

### 5.2. Definition of Qos Metrics

QualiCare processes calculate individual functions to extract metrics measures depending on the component role, which the QualiCare Manager gathers in its monitoring procedure. Table 4 shows these functions and the corresponding metrics. Components that calculate metrics CPU Load and Memory Load compute them for each process. The Data Acquisition Controller process computes the metrics Latency, Sampling Rate, and Transmission Rate for each Sensor Data Producer connection. In turn, the Data Access Handler process computes Jitter, Sampling Rate, and Transmission Rate for the sensor data transmitted for each user application connection.

### 5.3. Definition of Qos Services

QualiCare provides three different services for each QualiCare process depending on monitoring aspects: (*i*) resource elasticity; (*ii*) data compression; and (*iii*) parameters adaptation. The services are represented by the functions Elast(), Compress(), and Adapt(), respectively. Services can be provided individually or combined depending on the middleware status. QualiCare might provide each service through the different architecture components. Compress() is provided only at the Sensor level since it is the source of the data.

The Elast() service employs vertical elasticity strategies to increase or decrease the CPU and memory capacities without impacting in the processes operation. In particular, as the architecture modules are running in computing nodes, which might be physical or virtual machines, the service delivers new computing power to these instances. Elasticity is a popular concept in cloud platforms, which refers to the capacity of a system to automatically provision and de-provision resources according to workload changes [64]. QualiCare focuses on the vertical model of elasticity since it does no impose the modules to deal with duplicate instances which require load balancing strategies.

The Compress() service focuses on improving the network performance of nodes running the QualiCare processes. However, it might increase the need for more computing resources, resulting in the need for the Elast() service. Compress() is available in the Sensor Data Producer, and Data Acquisition Controller processes. These components employ compression algorithms to compress sensor data before packing it for transmission. This feature can be enable or disabled through a parameters which is part of the configuration of these modules. Enabling it allows the modules to apply the compression before sending it over the network. This decreases the network traffic, which improves communication between processes. Depending on some situations, the amount of data requested for a module can be higher than the transmission capacity of the module. Thus, compressing data is a possible way to decrease the amount of data and guarantee that requests are replied.

Finally, the Adapt() service consists of changing processes parameters, which affect the middleware behavior. Through this strategy, it is possible to change the sampling rate of data extraction from sensors or data acquisition the middleware performs. Additionally, it is also possible to define the data size in the Sensor level so that the number of bytes to be transmitted decreases. These adaptations provide hardware utilization improvements and modules parameters balancing. For instance, if modules are working on different sampling rates the Data Acquisition Controller process may request information in a lower sampling rate than a particular Sensor Data Producer process is generating. Decreasing the sampling rate of the Producer avoids wasting resources. In particular, when changing the sampling rate, each parameter adaption evaluates if the new configuration violates a upper or lower threshold. The threshold will be introduced in Section 5.5.1.

### 5.4. Qualicare Manager

**Figure 7:**
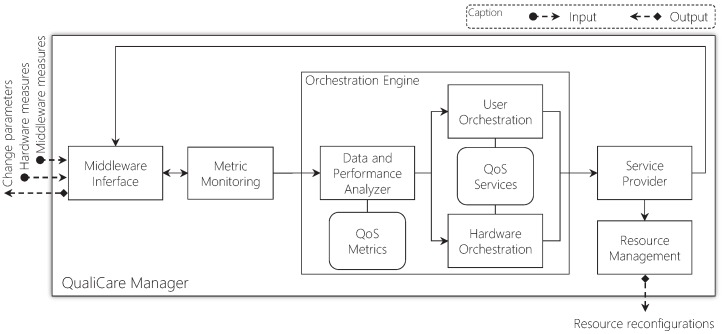
QualiCare Manager components, inputs, and outputs. The module receives measurements collected from the middleware and evaluates them based on metrics and services. The output are resource and parameters reconfigurations, if necessary, to guarantee QoS.

Figure 7 details the components of the QualiCare Manager depicting the inputs, and outputs. The Middleware Interface interacts with the middleware to collect metrics and send updates. Metric Monitoring is in charge of collecting each QoS metric measurement periodically at a given time interval. Thus, the main component, called Orchestration Engine, analyzes these measurements, including the feedback of previous decisions by comparing the variations in metrics measurements. The Engine contains the main strategies that the Manager applies to adapt the middleware. The Data and Performance Analyzer evaluates the QoS metrics from the middleware comparing them with QoS requirements to generate violation events. Thus, the User and Sensor Orchestration define actions that might be necessary to apply to tackle these events. After defining the actions, the Engine calls the Service Provider component to deliver the needed services. This component is able to call either the Middleware Interface, to provide the Adapt() and Compress() services, or the Resource Management to deliver the Elast() service.

**Figure 8:**
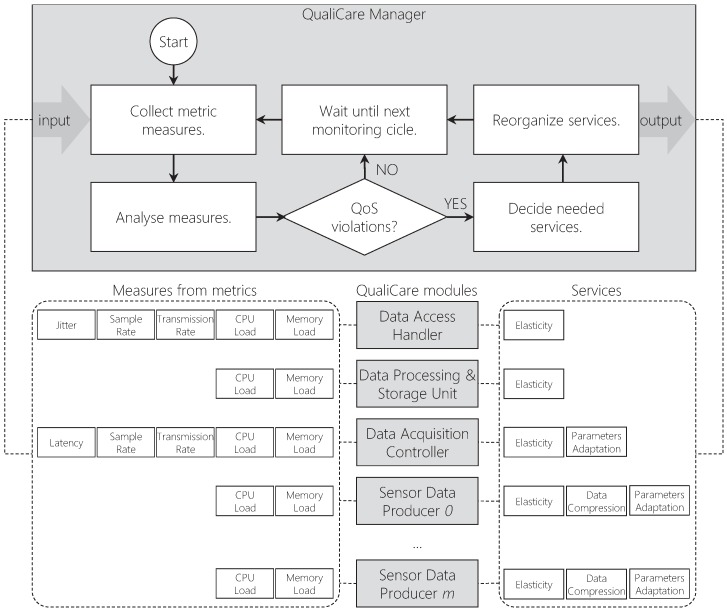
QualiCare Manager main monitoring cycle showing the service distribution and the metrics it evaluaves. The idea is to monitor module metrics and organize services according to the measures.

The QualiCare Manager process is in charge of monitoring metrics and delivering services for the different module processes. Each process has particular metrics and services that the Manager evaluates individually. Figure 8 depicts this task showing the main monitoring cycle. Additionally, Algorithm 1 details the Manager operations and procedures that occur periodically. First, the procedures from lines 4 and 5 collect data from the middleware and compute the QoS metrics. Then, the Orchestration Engine, through SensorOrchestration() and UserOrchestration(), define the actions the Service Provider must perform to tackle QoS violations through the procedure ProvideServices().

**Algorithm 1** QualiCare Manager Main Tasks.
**Input**: measures from metrics.
**Output**: middleware adaptions.
1:running ← true;2:cycle ← 0;3:**while** running **do**4:    CollectMonitoringData();5:    ComputeMetrics(qos_metrics,cycle);6:    sensor_orchestration_actions[] ← SensorOrchestration(qos_metrics,qos_services);7:    user_orchestration_actions[] ← UserOrchestration(qos_metrics,qos_services);8:    **if** sensor_orchestration_actions > 0 **then**9:        ProvideServices(sensor_orchestration_actions);10:        sensor_orchestration_actions.clear();11:    **end if**12:    **if** user_orchestration_actions > 0 **then**13:        ProvideServices(user_orchestration_actions);14:        user_orchestration_actions.clear();15:    **end if**16:    sleep();17:    cycle++;18:
**end while**



### 5.5. Service Orchestration

**Figure 9:**
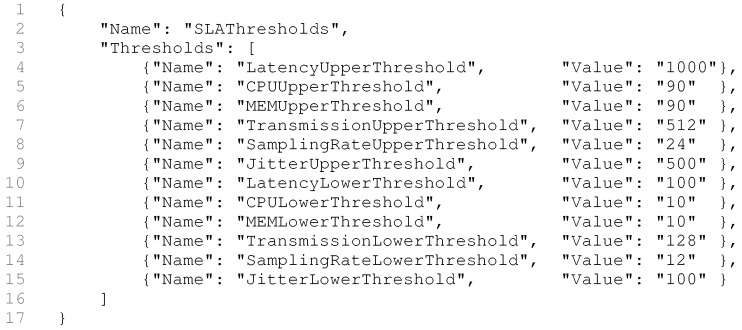
SLA file containing the default values for each threshold used in the Orchestration Engine algorithms.

In cloud computing environments, resource orchestration consists of a set of operations that cloud providers offer to dynamically adjust hardware and software resources to guarantee QoS [65]. Deriving from these concepts, the QualiCare service orchestration is a decision process that selects and delivers the stack of services for each user application and sensor connected to the middleware. QualiCare adopts a rule-based strategy to choose which services are suitable to address QoS situations. This solution follows a Service Level Agreement (SLA) strategy which consists of a set of rules that monitors limits, called thresholds, for a given metric. SLA-base strategies are common in self-adaptable solutions which employ rule-based strategies, as in Hanif et al. [66]. By starting the QualiCare Manager, it receives as input an SLA file in a JSON format, according to the RFC 2647 [67], containing the default rules and thresholds for each metric. It is possible to change the behavior of the system just by editing the SLA file and running the QualiCare Manager again. Figure 9 demonstrates an SLA file example containing the values for the thresholds of the model. The file defines the name of the threshold and its value.

**Figure 10:**
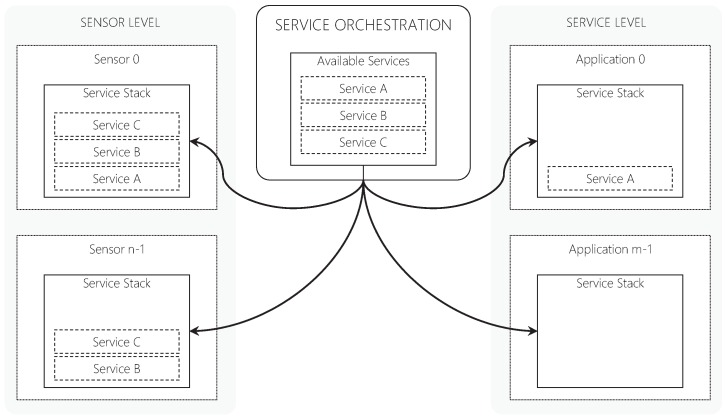
Example of the service orchestration process performed by the Orchestration Engine.

At each monitoring cycle, the Data and Performance Analyzer component accesses the current measures for each module/connection and evaluates them against the upper and lower thresholds by a multi-level rule set. Therefore, the service orchestration is performed for both User and Sensor levels. The User Orchestration and Sensor Orchestration define the final stack of services for each module based on the results of the threshold analysis. Figure 10 depicts an example of the multi-level orchestration model showing that user applications and sensors have individual service stacks. QualiCare adopts a rule-based strategy to choose which service(s) is suitable to address situations, which impact the middleware QoS. At each monitoring cycle, the Data and Performance Analyzer accesses the current measures for each module/connection and evaluates them against upper and lower thresholds by a multi-level rule set. The User Orchestration and Sensor Orchestration component define the final stack of services for each module based on the results of the threshold analysis.

#### 5.5.1. Sensor Orchestration

In the Sensor Orchestration, the Engine manages the service stack for each Producer individually. One main rule, based on the $Lat(s_{i})$ metric, and a set of sub-rules, one for each available service, compose the rule-based approach. After collecting all metrics from the connected producers, for each Producer $s_{i}$, the Engine verifies if its current latency $Lat(s_{i})$ is violating an upper threshold or a lower threshold. It uses the latency metric since a high latency when acquiring data from sensors causes delays on delivering sensor data to the database and user applications that are expecting such information. Therefore, if the latency of a Sensor Data Producer is violating the upper threshold, the Engine evaluates three different metrics to choose between services to deliver: (*i*) high CPU and Memory loads, which may delay the processing of requests; (*ii*) high transmission rate, which can identify network congestion; and (*iii*) high sampling rate, which may cause high processing loads and network congestion. On the other hand, if the latency is below a given lower threshold, then the same metrics are evaluated to verify if they also may be violating a lower threshold. When violating lower thresholds, it is possible to release resources or increase the sampling rate to produce more data which results in better resource utilization. Algorithm 2 details the main operations the Orchestration Engine performs to orchestrate services in the Sensor level. This algorithm corresponds to the SensorOrchestration(qos_metrics,qos_services) function from Algorithm 1 (line 6). Lines 3 and 14 compose the main rules, which the algorithm checks for each $s_{i}$ Sensor Data Producer process. The service_list stores the actions that the function returns, which the Service Provider delivers.

**Algorithm 2** Sensor Orchestration Exectution Tasks.
**Input**: SLA threshold file and metrics measures.

**Output**: QoS services.
1:service_list ← new empty list;2:**for** each $s_{i}$ Sensor Data producer process **do**3:    **if** $Lat(s_{i})$ > LatencyUpperThreshold **then**
4:        **if** $CPU(p_{q})$ > CPUUpperThreshold & $MEM(p_{q})$ > MEMUpperThreshold **then**
5:           service_list.add($s_{i}$,Elast(“increase”));6:        **end if**7:        **if** $Tra(a_{i})$ > TransmissionUpperThreshold **then**
8:           service_list.add($s_{i}$,Compress());9:        **end if**10:        **if** $Sam(a_{i})$ > SamplingRateUpperThreshold **then**
11:           service_list.add($s_{i}$,Adapt(“decrease”));12:        **end if**13:    **end if**14:    **if** $Lat(s_{i})$ < LatencyLowerThreshold **then**
15:        **if** $CPU(p_{q})$ < CPULowerThreshold & $MEM(p_{q})$ < MEMLowerThreshold **then**
16:           service_list.add($s_{i}$,Elast(“decrease”));17:        **end if**18:        **if** $Tra(a_{i})$ < TransmissionLowerThreshold **then**
19:           service_list.add($s_{i}$,Compress());20:        **end if**21:        **if** $Sam(a_{i})$ < SamplingRateLowerThreshold **then**
22:           service_list.add($s_{i}$,Adapt(“increase”));23:        **end if**24:    **end if**25:
**end for**
26:**return** service_list;

$s_{i}$: a Sensor Data Producer process;
$p_{q}$: the QualiCare process identification corresponding to $s_{i}$;
$a_{i}$: a user application or Sensor Data Producer process;


#### 5.5.2. User Orchestration

In the User Orchestration, the Engine employs a different set of rules. Algorithm 3 defines the operations the user orchestration performs in its execution process. This algorithm corresponds to the function UserOrchestration(qos_metrics,qos_services) from Algorithm 1 (line 7). Each user application may define its QoS requirements with respect to jitter. The requirements define upper ($JT_{u}$) and lower ($JT_{l}$) thresholds for the metric $Jit(u_{j})$, which is used in the main rules of the model. If the application does not provide its requirements, the thresholds are initialized with default values. In contrast to the Sensor Orchestration which uses the latency as the main metric, in the User Orchestration the Engine verifies, for each user application connection $u_{j}$, if its current jitter $Jit(u_{j})$ is violating an upper threshold or a lower threshold. It uses this metric since a high jitter when acquiring data from sensors causes instabilities and delays on delivering sensor data to the applications. Therefore, if the jitter of a $u_{j}$ user connection managed by the Data Access Handler is violating the upper threshold, the Engine verifies two different metrics to choose between services to deliver: (*i*) high CPU and Memory loads, which causes delays on processing the replies to be sent to the applications; and (*ii*) high transmission rate, which causes network congestion if the number of applications is high. The same metrics are evaluated to verify if they also may be violating a lower threshold. When violating lower thresholds, the Engine may decrease the allocated resources and deactivate compression algorithms which may decrease response time to requests. As in the sensor orchestration algorithm, the service_list stores the actions the function returns to the Service Provider.

**Algorithm 3** User Orchestration Execution Tasks.
**Input**: SLA threshold file and metrics measures.

**Output**: QoS services.
1:service_list ← new empty list;2:**for** each $u_{j}$ user application **do**3:    **if** $Jit(u_{j})$ > JitterUpperThreshold **then**
4:        **if** $CPU(p_{q})$ > CPUUpperThreshold & $MEM(p_{q})$ > MEMUpperThreshold **then**
5:           service_list.add($u_{j}$,Elast(“increase”));6:        **end if**7:        **if** $Tra(a_{i})$ > TransmissionUpperThreshold **then**
8:           service_list.add($u_{j}$,Compress());9:        **end if**10:    **end if**11:    **if** $Jit(u_{j})$ < JitterLowerThreshold **then**
12:        **if** $CPU(p_{q})$ < CPULowerThreshold & $MEM(p_{q})$ < MEMLowerThreshold **then**
13:           service_list.add($u_{j}$,Elast(“decrease”));14:        **end if**15:        **if** $Tra(a_{i})$ < TransmissionLowerThreshold **then**
16:           service_list.add($u_{j}$,Compress());17:        **end if**18:    **end if**19:
**end for**
20:**return** service_list;

$u_{j}$: a user application connection;
$p_{q}$: the QualiCare process identification corresponding to $u_{j}$;
$a_{i}$: a user application or Sensor Data Producer process;


## 6. Results and Discussion

The model evaluation methodology consists on deploying the middleware in a simulated OR at the Unisinos Softwarelab (http://www.unisinos.br/softwarelab/en/) and performing experiments with the Sewio indoor RTLS solution (https://www.sewio.net/real-time-location-system-rtls-on-uwb/). We installed in the room a wired Gigabit Ethernet network, and a computing node (4GB RAM, quad-core Core i5) running a Sensor Data Producer instance acquiring tag positions from the RTLS system at 30 FPS. We are using the computing node with such configuration since in future works we are planning to employ Computer Vision strategies and perform experiments that consider the extraction of depth and color images from camera devices to estimate human poses. We consider this kind of experiments due to these strategies be present in healthcare environments [68]. We deployed the remaining QualiCare components, including the QualiCare Manager, in an additional server node. At this research stage, we are evaluating the Sensor Data Producer performance and communication. By combining the number of tags in the room and the number of connections the Producer receives, we designed four different execution scenarios with 10 min of duration:

1. 1 tag with 1 connection (1T1C);
2. 1 tag with 3 connections (1T3C);
3. 3 tags with 1 connection (3T1C);
4. 3 tags with 3 connections (3T3C).

Additionally, the same scenarios were executed with QualiCare Manager delivering the Adapt() service (Algorithm 2, line 11) decreasing the FPS by half. Although this can decrease the sampling rate a user application may be expecting, the Adapt() service always respect the SamplingRateLowerThreshold value so that the minimum FPS is the value of this threshold. In our experiments, as we are evaluating the Sensor Data Producer performance and communication, and as we are starting from an FPS equal to 30, we defined the SamplingRateLowerThreshold to 15 FPS.

**Figure 11:**
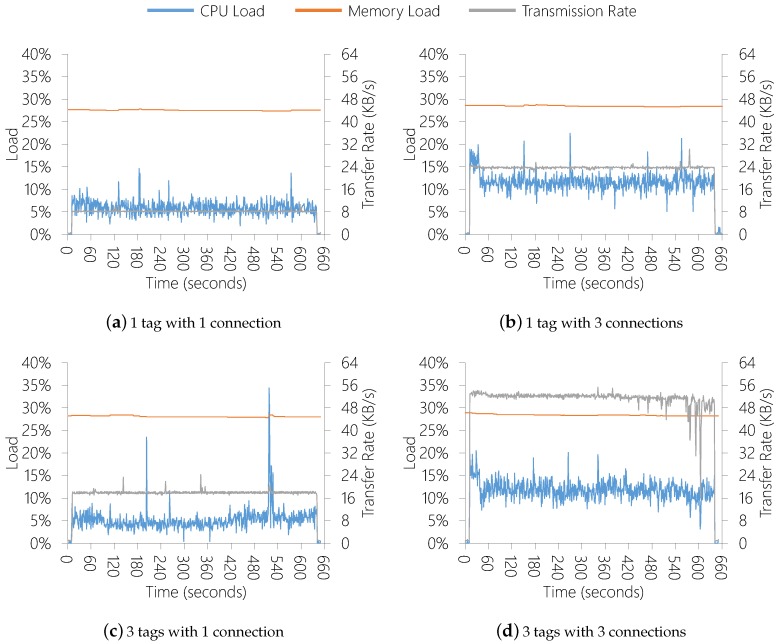
Results without parameters adaptation.

Figure 11 depicts the results without performing adaptions in the FPS. The network load is highly impacted by changing the number or connections or tags since the amount of data to be transmitted increases in these scenarios. Additionally, the processing load suffer more impact serving more clients than extracting more tag positions from the room. On the other hand, memory load does not suffer critical impacts in all scenarios. It occurs due to the size of information from tags being low, compared with the available memory.

**Figure 12:**
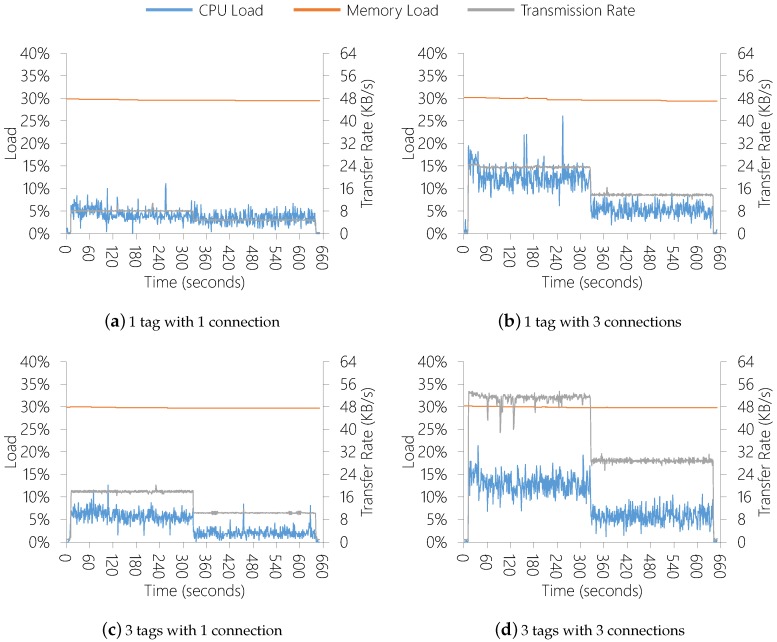
Results with QualiCare Manager adapting the FPS.

Figure 12, on the other hand, depicts the results with QualiCare Manager adjusting the FPS. In all graphs, it is possible to visualize the impact of the adaption, which occurs in the second 330. This demonstrates that changing FPS from Sensor Data Producer instances has two main advantages. First, the hardware utilization is improved, which might also impacts in energy consumption. Second, it is possible to serve more clients simultaneously since the adaption frees network and processing to new connections.

## 7. Limitations

It is worth noting some limitations which might be explored in future research. Currently, the Orchestration Engine from the QualiCare Manager defines a threshold rule-based strategy in the user and sensor levels of the middleware. We intend to change the service orchestration strategy by a different heuristic to select the services for user applications and sensors. On the other hand, we also intend to adapt the current strategy so that the user can also configure the system rules through an SLA configuration file like the SLA threshold file. Moreover, we are considering explore the effect of the previous decision by comparing the variation in metrics measurements. This feedback allows the employment of techniques to learn from previous actions and consider past events in the decision-making process. Considering the services, it is possible to explore the horizontal model of resource elasticity. In this regard, load balancing strategies and new strategies for communication reconfiguration are the main challenges.

At the current stage of this research, we have evaluated only the Sensor Data Producer module with the sampling rate metric. The next steps include the evaluation of the remaining modules and metrics in a real OR environment. Currently, we are deploying the infrastructure in a OR so that we can conduct more detailed experiments. Although we do not present deep evaluation of the prototype, our current results demonstrate the impact in network and processing resources. In future work, we also intend to evaluate the model with data from larger health databases and focus on increasing the possibilities of benefits for patients and healthcare providers. Other important aspects to discuss are data distribution, scalability, security, and privacy. In addition, the prototype can expand to integrate with other open and proprietary health standards.

## 8. Conclusions

Many different sensors are in charge of monitoring subjects in the OR, which might be things or persons. Since data from surgical procedures are critical, its continuous flow is essential to avoid critical situations and evaluation of the room setup. In addition, as the OR represents one of the highest costs in hospitals, improving procedures efficiency is also important. Several research focus OR environments proposing medical and monitoring systems to improve service performance and increase patient safety. The literature also presents developments of IoT middlewares which support QoS in several fields. However, they do not present the combination of QoS middlewares for OR environments, which demand monitoring of several equipment and people.

In this context, this article presented an adaptable middleware model to provide multi-level QoS on ORs called QualiCare. The scientific contributions of this research are twofold: (*i*) we propose a model of an adaptable QoS-enabled middleware for OR; and (*ii*) we define an orchestration engine to provide different services for user applications and sensors. The model offers a set of services to guarantee the middleware scalability regardless the number of sensors and user applications producing and consuming data, respectively. QualiCare guarantees that one or more applications consume data from the middleware turning it scalable according to the applications load. QualiCare acts in both user applications and sensors levels in order to guarantee real-time data. To offer these features, QualiCare employs a rule-based service orchestration process based on a lower and upper thresholds. QualiCare monitors specific metrics in the different levels to combine its measures in the orchestration process for decision-making. Additionally, the model encompasses a Manager process which is charge of QoS monitoring and service provisioning. This component performs parameters adaptation, compression, and vertical elasticity in the middleware resources to tackle QoS violations. Besides the scientific contributions, this research also presents a social contribution related to the patients safety. By guaranteeing QoS on delivering medical information for hospital administrators and physicians, QualiCare ensures that sensitive data is properly delivered to its destinations regardless the system load. This might improve medical outcomes and consequently save lives.

Results show that adapting the sampling rate of sensors within acceptable levels decreases the transmission rate consequently. Although our evaluation is preliminary, the experiments are promising and demonstrate the system significance, even more considering hospitals with many surgical rooms and long duration procedures. It is worth noting some limitations which might be explored in future research. In future work, we intend to evaluate the model running the Sensor Data Producer instances in a private virtual environment which enables replication and different elasticity models. This will require load balancing strategies and new strategies for communication reconfiguration in the service and sensor levels.

## Figures and Tables

**Table 1:** Summary of articles resulting from the review methodology.

Focus	Reference	Year	Middleware	Real-Time	Goal	Area	Technologies	Description
Patient monitoring	[41]	2013	✓		Monitoring	WSN	Zigbee	Telemedicine
	[39]	2017			Tracking	RTLS	RFID	Postoperative abdominal surgery
	[40]	2017			Monitoring	IoT	Pub/Sub	Ambient assisted living
	[38]	2017	✓	✓	Tracking	RTLS	RFID	Medication error monitoring
	[42]	2018			Tracking	IoT	RFID	Hospital facilities
Device Integration and Data Interoperability	[43]	2010	✓	✓	Monitoring	-	Pub/Sub	Hospital device framework
	[44]	2012			Monitoring	CPS	Pub/Sub	Device integration and data extraction
	[47]	2012	✓		-	-	Pub/Sub	Medical applications
	[48]	2014	✓	✓	Communi- cation	Medical Systems	LAN	Eye surgery
	[46]	2016		✓	Monitoring	RTPS	Pub/Sub	Medical devices integration
	[49]	2016	✓	✓	-	IoT	Pub/Sub	Data exchange
	[45]	2017	✓		Monitoring	IoT	-	Integrated clinical environment
Smart Hospitals	[50]	2012	✓		Tracking	Ubiquitous Computing	RFID	Hospital location and tracking
	[51]	2015		✓	Tracking	-	RFID	Review of concepts
	[52]	2016	✓	✓	Tracking	RTLS	RFID	Ward management
Operating Room Monitoring (Intelligent OR)	[24]	2010	✓	✓	Monitoring	WSN	Sensors	Sensor data acquisition in minimally invasive surgery
	[19]	2011		✓	Activity recognition	Ubiquitous Computing	-	Recommendation system for surgical procedures
	[25]	2010	✓	✓	Tracking	RTLS	RFID	Medical error detection system
	[26]	2011	✓	✓	Tracking	RTLS	RFID	Literature review and system for surgical sponges and personnel tracking in surgery
	[28]	2011	✓	✓	Tracking	RTLS	RFID	Patient workflow monitoring in surgeries
	[21]	2011	✓	✓	Tracking	Computer Vision and RTLS	Proprie- tary trackers	Neurosurgery tracking from heterogeneous sources
	[20]	2011	✓	✓	Tracking/ activity recognition	RTLS	RFID	Surgical monitoring system to identify critical situations
	[23]	2012	✓	✓	Tracking	RTLS	RFID	Surgical monitoring system to identify critical situations
	[27]	2012	✓	✓	Tracking	RTLS	RFID	OR team and sponge real-time tracking
	[22]	2014	✓	✓	Tracking	RTLS	RFID	Patient tracking and surgical workflow monitoring
	[18]	2017			Activity recognition	Computer Vision	RGB-D Camera	Human pose estimation and activity recognition during surgery
QoS-Aware Middlewares	[53]	2018	✓		Network monitoring	Edge Computing	MQTT	Distributed QoS-Aware MQTT middleware for Edge Computing applications
	[54]	2018	✓		Network traffic	IoT	-	QoS management modules
	[55]	2018	✓		Network traffic	IoT	-	QoS management modules
	[56]	2019	✓		Network monitoring	IoT	Pub/Sub and SDN	Differentiation of services in SDN-like Pub/Sub middlewares for IoT
	[57]	2019			Literature Review	IoT	-	Systematic literature review

**Table 2:** Description of the network header fields.

Field	Description
Type	The payload type.
ID	Identification of the request.
Payload Size	The size of the payload in bytes.
Remaining	Number of messages remaining to answer the request.

**Table 3:** Description of the data attributes from sensor data packages.

Field	Description
Type	The data type.
Timestamp	The time in milliseconds that the data is collected from the sensor.
Sample Count	The sequence number of the collected data.
Data Producer ID	The identification of the module that collected the data.
Device ID	The identification of the device from which the data was collected.
Sensor ID	The identification of the sensor from which the data was collected.

**Table 4:** QoS metric definition and their corresponding functions.

Metric	Function	Source
Latency	$Lat(s_{i})$	Data Acquisition Controller
Jitter	$Jit(u_{j})$	Data Access Handler
Sampling Rate	$Sam(a_{n})$	Data Acquisition Controller and Data Access Handler
Transmission Rate	$Tra(a_{n})$	Data Acquisition Controller and Data Access Handler
CPU Load	$CPU(p_{q})$	All components
Memory Load	$MEM(p_{q})$	All components
